# Medical students’ attitudes toward interactions with the pharmaceutical industry: a national survey in Japan

**DOI:** 10.1186/s12909-018-1394-9

**Published:** 2018-12-04

**Authors:** Sayaka Saito, Takami Maeno, Yasushi Miyata, Tetsuhiro Maeno

**Affiliations:** 1 Department of Primary Care and Medical Education, Graduate School of Comprehensive Human Sciences, University of Tsukuba, 1-1-1 Tennodai, Tsukuba, Ibaraki 305-8575 Japan; 2 Department of Primary Care and Medical Education, Faculty of Medicine, University of Tsukuba, 1-1-1 Tennodai, Tsukuba, Ibaraki, 305-8575 Japan; 3 Department of Primary Care and Community Medicine, Medical Education Center, Aichi Medical University School of Medicine, 1-1 Yazakokarimata, Nagakute, Aichi 480-1195 Japan; 4 Department of Primary Care and Medical Education, Faculty of Medicine, University of Tsukuba, 1-1-1 Tennodai, Tsukuba, Ibaraki 305-8575 Japan

**Keywords:** Medical students, Pharmaceutical industry, Conflict of interest, Undergraduate education, Medical education

## Abstract

**Background:**

The relationship between students and the pharmaceutical industry has received substantial attention for decades. However, there have been few reports on this issue from East Asia. We aimed to investigate Japanese medical students’ interactions with and attitudes toward the pharmaceutical industry, and to assess the correlation between exposures to a formal curriculum on drug promotion and perceptions of the appropriateness of the physician–industry relationship.

**Method:**

We invited all 80 medical schools in Japan to participate. A cross-sectional anonymous survey was administered to medical students and school staff at the 40 schools that participated. The questionnaire for students assessed interactions with and attitudes toward the pharmaceutical industry. The questionnaire for school staff assessed the formal undergraduate curriculum.

**Results:**

Forty of the 80 medical schools in Japan participated. The response rate to the medical student survey was 74.1%, with 6771 evaluable responses. More than 98% of clinical students had previously accepted a small gift of stationery, a brochure, or lunch, and significantly higher percentages of clinical than preclinical students had accepted one or more gifts (*P* < .001). Among preclinical and clinical students, respectively, 62.7 and 71.9% believed it was appropriate to accept stationery, and 60.5 and 71.0% thought that attending an industry-sponsored lunch did not influence clinical practice. Of the 40 participating schools, 13 (33.0%) had a formal curriculum on drug promotion. A multivariate analysis showed an association between exposure to a formal curriculum and students’ perceptions of the appropriateness of the physician–industry relationship only for gifts of stationery, which were perceived as inappropriate (OR: 0.81, 95% CI: 0.69–0.95, *P* = .02).

**Conclusions:**

Most Japanese medical students interact with the pharmaceutical industry and believe that gift acceptance is appropriate and not influential. This study demonstrated a limited association between students’ perceptions of gift appropriateness and exposure to a formal curriculum.

## Background

The physician–industry relationship has received substantial attention for decades. Studies from many countries show that physician–industry interaction is common, and that this interaction can lead to higher prescribing costs, lower prescribing quality, and lower patient trust in physicians [1–5]. In the United States, the Institute of Medicine (IOM), the American Medical Association (AMA), and the American College of Physicians (ACP) released stringent ethical codes in 2008, and the Pharmaceutical Research and Manufacturers of America (PhRMA) revised their promotion code in 2009 [6–10]. The Physician Payment Sunshine Act was also proposed in 2009 [11]. A national survey in the United States indicated that interactions between physicians and the pharmaceutical industry changed after these regulatory initiatives; physician meetings with sales representatives decreased from three to two per month, and the percentage of physicians who received any gifts decreased from 83 to 71% [12].

Reports from several countries have shown that medical students also frequently interact with the pharmaceutical industry [13–17]. In the United States, the Association of American Medical Colleges (AAMC) released a task force report in 2008 calling on academic health centers to develop rules for interactions with the industry, as well as educational programs to teach physicians and students about this relationship [18]. A follow-up survey in eight medical schools in the United States showed that the number of interactions between medical students and the industry decreased from 4.1 per month in 2003 to 1.6 per month in 2012, and the percentage of medical students who received any gifts decreased from 100 to 79% [17]. Further, the percentage of students who agreed with the statement “It is sometimes okay for medical students to accept gifts from drug companies because drug companies have minimal influence on students” decreased from 71% in 2003 to 39% in 2012. Similarly, the percentage of students who believed that information is biased if it is presented in grand rounds sponsored by drug companies increased from 67% in 2003 to 83% in 2012. Although students in 2012 generally had a more skeptical attitude toward the pharmaceutical industry than in 2003, many still had a favorable attitude, and 67% of students thought that information from the industry was useful.

Despite Japan being the second-largest pharmaceutical market in the world and the expanding pharmaceutical market in other East Asian countries, there have been few reports on this issue from East Asia. In Japan, we reported that Japanese physicians met with sales representatives seven times per month and that 96% of them received a small gift of stationery (such as a pen) and 49% accepted industry-sponsored meals outside their workplace [19]. In addition, a national survey of Japanese medical students in 2012 reported that 37% of preclinical and 98% of clinical students had received stationery such as pens and notepads, and 21% of preclinical and 97% of clinical students had received a lunchbox at promotional meetings for pharmaceutical industry products [20]. However, attitudes toward interactions between medical students and the pharmaceutical industry in East Asian countries have never been evaluated. Moreover, there’s no report about to what extent Japanese medical students have an opportunity to learn about physician-industry relationships.

We conducted a cross-sectional survey to investigate Japanese medical students’ interactions with and attitudes toward the pharmaceutical industry. We also examined the undergraduate curriculum on drug promotion in Japanese medical schools and assessed the association between students’ attitudes and exposure to a formal curriculum.

## Methods

We sent the deans of all 80 medical schools in Japan a request form for study participation between April and May 2016. A cross-sectional survey consisting of separate questionnaires for (I) medical students and (II) school staff members (regarding the formal curriculum) was conducted during a single study period. The institutional review board at Tsukuba University approved the survey protocol.

(I). **Survey of medical students**

### Participants

The target population consisted of all medical students in Japan. Preclinical and clinical students in medical schools whose deans approved study participation were enrolled. In general, medical students receive education for 6 years in Japan, and are offered a general liberal arts education for the first 2 years, lectures in basic medicine before clinical practice in the next 2 years, and clinical practical training in the last 2 years [21]. Preclinical students had not yet begun their clinical clerkship programs, and the persons in charge at each school selected the school year in which it would be feasible to distribute and collect the questionnaire within the research period as the participating year. Clinical students in Japan are in their fifth and sixth years in medical school. The 11-month investigation period ran from the beginning of a school year to the end of a school year. Thus the inclusion of fifth-year students in the study would have meant that some participants had just started clinical training, whereas others had experienced clinical training for almost 1 year. We assumed that the less clinical training, the larger the impact of the clinical training duration on the degree of exposure to the industry. Therefore, we included only sixth-year clinical students, as these had all experienced a year of clinical training.

### Medical student survey instrument

A 23-item, four-page anonymous questionnaire was developed after a literature review and discussion among the authors [13, 15, 17, 19, 20, 22–25]. The questionnaire was not pilot-tested before use. The questionnaire assessed students’ background information as well as their interactions with and attitudes toward the pharmaceutical industry. The cover page stated the purpose of the study and confirmed the voluntary nature of participation and the confidentiality of responses. No incentives were offered for participation.

#### Interactions with industry

Of the 15 items that assessed exposure to the pharmaceutical industry in the survey administered to Japanese medical students in 2012 [20], five items were also included in a previous survey of Japanese physicians [19]. These questions asked if participants had ever accepted or attended, as appropriate, the following:

- Stationery, such as a pen or notepad
- A medical textbook or a book of clinical practice guidelines
- A brochure of a pharmaceutical company’s products
- A lunch provided at a promotional meeting about a company’s products
- A seminar, workshop, or lecture sponsored by a pharmaceutical company

#### Attitudes toward interactions with industry

**Fig. 1:**
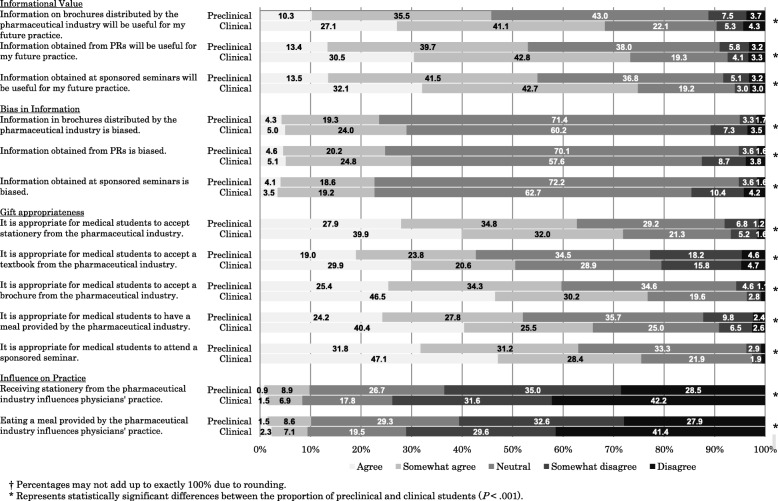
Medical students’ attitudes toward the physician-pharmaceutical industry relationship

We asked to what extent students agreed with 13 statements about “informational value,” “bias in information,” “gift appropriateness,” and “influence on practice” using a 5-point Likert scale (disagree, somewhat disagree, neutral, somewhat agree, or agree) (Fig. 1). Three items related to “informational value” and asked whether students thought information from brochures, from sales representatives, and in seminars was useful for their future practice. Three items related to “bias in information” and asked whether students thought information from brochures, from sales representatives, and in seminars was biased. Five items related to “gift appropriateness” and asked whether students thought it was appropriate for medical students to accept stationery or textbooks, brochures, or lunch, or to attend a seminar. The other two items related to “influence on practice” and asked whether students thought that accepting stationery and lunch influenced physicians’ clinical practice.

#### Backgrounds of participants

In addition to sex, age, and years in medical school, we asked participants whether either of their parents were medical doctors. We also asked if they had previously received any teaching on conflicts of interest related to interactions with the pharmaceutical industry (prior teaching on the physician–pharmaceutical relationship).

### Medical student survey administration

Between May 2016 and March 2017 we sent a set of questionnaires to the staff or faculty member specified by the dean of each of the 40 participating schools. Questionnaires were distributed in a class with required attendance and collected on that occasion. Each institution was allowed to choose when to distribute and collect questionnaires. Leaflets notifying students of the current regulations regarding physician–industry relationships were distributed just after the questionnaires were collected to relieve any potential unease caused by students’ lack of knowledge of physician–industry relationships. Completed questionnaires were returned by mail.

(II). **Survey on undergraduate formal curriculum on drug promotion**

A questionnaire about the undergraduate formal curriculum on drug promotion was sent to the staff or faculty member specified by the dean of each participating school; this was sent separately from the set of student questionnaires. We asked whether the school offered a formal curriculum on drug promotion. If respondents answered “Yes” to this question, we asked whether the course was required or elective, and during what year students took the course. If the questionnaire responses did not provide sufficient information on whether the class being surveyed (I) was exposed to such a course, we asked for confirmation from the person in charge.

### Analysis

A questionnaire was considered to be evaluable if it was returned by the pre-specified deadline (March 31, 2017) and contained complete information for at least 80% of the 23 items. Multiple responses to a question for which a single response was appropriate were considered void and were not evaluated. To ensure the completeness and accuracy of data entry, the first author (SS) entered the data into two separate Microsoft Excel datasheets. The two datasets were compared and data entry errors were corrected. SPSS version 23.0 (IBM Corp: Armonk, NY) was used for all statistical analyses.

Descriptive statistics were calculated for all variables. The calculation of the percentages reflects the fact that the denominators for each question differed according to the number of valid responses. We used Pearson’s chi-square tests to compare preclinical and clinical students in terms of the association between responses to each item and students’ background information, interactions with the pharmaceutical industry, and attitudes to industry gifts. Multivariate logistic regression models (simultaneous method) were used to assess predictive factors associated with students’ attitudes to the appropriateness of pharmaceutical industry gifts. These attitudes were the dependent variable and were dichotomized into appropriate/somewhat appropriate and somewhat inappropriate/inappropriate/neutral (the latter was the reference category). The following independent variables were used: students’ background characteristics (sex, type of school, whether either parent was a medical doctor, prior teaching on the physician–pharmaceutical relationship); exposure to a formal curriculum on drug promotion as reported by school staff in survey (II); each type of interaction with the pharmaceutical industry; and attitudes toward each interaction in terms of informational value, bias in information, and influence on practice. We excluded age, school year, and clinical status (preclinical/clinical), because these variables were highly correlated with interactions. We dichotomized schools into national government-funded (national)/local government-funded (prefectural) (as the reference category) and private. This was because ethical codes to restrict gifts from interested parties are applied in national and prefectural universities, but not in private universities. Based on the results of bivariate analysis, students’ attitudes were dichotomized as follows, with the second category in each case serving as the reference: agree/somewhat agree and neutral/somewhat disagree/disagree with the statements if the information from three sources are useful for “informational value”; not biased/not very biased and neutral/somewhat biased/biased for “bias in information”; and not influential/not very influential and neutral/somewhat influential/influential for “influence on practice.” For example, in the multivariable model for appropriateness of stationery, the independent variables were background characteristics, prior acceptance of stationery (yes = 1, no = 0), and influence of stationery on practice (not influential = 1, influential = 0). Odds ratios (ORs) with 95% confidence intervals (CIs) were calculated. For all statistical analyses, *P* values were 2-tailed and those less than .05 were considered statistically significant.

## Results

Forty (50%) of the 80 medical schools in Japan participated in the study. Of these, 22 were national (51.2% of all national schools), 6 were prefectural (75.0% of all prefectural schools), and 12 were private (41.4% of all private schools). Thirteen schools (32.5%) had formal curriculum on drug promotion (Table 1). Of 9132 students in the surveyed classes, 7029 (77.0%) responded to the questionnaire. There were 188 (2.1%) incomplete answer sheets containing answers to fewer than 80% of the 23 items; 40 (0.4%) were blank; and 30 (0.3%) responses from three classes were judged ineligible because the survey was not administered according to protocol. Thus there were 6771 evaluable responses and an overall response rate of 74.1%. The response rate of preclinical students was 72.8% (3395/4661) and that of clinical students was 75.5% (3376/4471).

**Table 1:** Characteristics of participating medical schools (*N* = 40)

		Number	%
Type	national	22	55
	prefectural	6	15
	private	12	30
Formal curriculum on drug promotion			
	yes	13	32.5
	no	27	67.5

### Respondent characteristics

The characteristics of the respondents are shown in Table 2. The mean age of preclinical students was 21.3 years (standard deviation [SD] = 2.81) and that of clinical students was 25.4 years (SD = 3.14). About two-thirds of both preclinical and clinical students were men. The percentage of respondents with at least one parent who was a medical doctor was approximately 40%; there was no significant difference on this between preclinical and clinical students. Of the preclinical and clinical students, 10.8 and 38.9%, respectively, reported that they had received teaching about conflicts of interest in the physician–industry relationship (*P* < .001), and 3.0 and 32.3% had been exposed to a formal curriculum on drug promotion, which was confirmed by school staff members. Of students who perceived that they had received education on conflicts of interest with the pharmaceutical industry, 10.6% (n/*N* = 39/367) of the preclinical group and 34.4% (450/1308) of the clinical group had been exposed to a formal curriculum on drug promotion. Of students who had been exposed to a formal curriculum on drug promotion, 38.6% (39/101) of the preclinical group and 41.2% (450/1091) of the clinical group perceived that they had received education on conflicts of interest with the pharmaceutical industry.

**Table 2:** Characteristics of respondents

		Preclinical (*n* = 3395)		Clinical (*n* = 3376)	
School year, n	1st	705			
	2nd	1101			
	3rd	1479			
	4th	110			
	6th			3376	
Age, mean (standard deviation)		21.3 (2.81)		25.4 (3.14)	
		n	%	n	%
Sex	male	2178	64.2	2160	64.1
	female	1214	35.8	1210	35.9
Type of school	national	1756	51.7	1819	53.9
	prefectural	438	12.9	364	10.8
	private	1201	35.4	1193	35.3
One or both parents were a medical doctor	yes	1245	36.7	1287	38.2
	no	2146	63.3	2081	61.8
Receiving prior teaching on the physician-industry relationship	yes	367	10.8	1308	38.9
	no	3019	89.2	2057	61.1
Exposure to a formal curriculum on drug promotion	yes	101	3.0	1091	32.3
	no	3294	97.0	2285	67.7

### Interaction with the pharmaceutical industry

Of the preclinical students, 1031/3393 (30.4%) had accepted stationery from the pharmaceutical industry. Fewer had experienced interactions involving the other four items: 5.3% for a textbook, 17.6% for a brochure, 13.0% for lunch, and 8.7% for a sponsored seminar. More than 98% of clinical students had received stationery, a brochure, or lunch; 80.1% (2703/3376) had attended a sponsored seminar; but only 26.7% (901/3370) had received a textbook. The percentage of students who had accepted gifts was significantly higher for each item in clinical students than in preclinical students (all *P* < .001) (Table 3).

**Table 3:** Proportions of Japanese medical students who had interacted with the pharmaceutical industry

	Preclinical (*n* = 3395)*		Clinical (*n* = 3376)*		Comparison of proportions, preclinical versus clinical✝
Type of gift or event	n	%	n	%	*P value*
Stationery	1031	30.4	3318	98.3	< .001
Medical textbook	181	5.3	901	26.7	< .001
Brochure	598	17.6	3328	98.6	< .001
Lunch provided at a promotional presentation	440	13.0	3332	98.8	< .001
Sponsored seminar	294	8.7	2703	80.1	< .001

✝Pearson’s chi-square test

*Sample size varied by item because of non-respondents. The proportion of non-respondents was less than .18%

### Attitudes toward interaction with the pharmaceutical industry

Students’ attitudes toward their relationship with the pharmaceutical industry are shown in Fig. 1.

- Informational value

Significantly more clinical than preclinical students thought that information from the industry was useful. For example, 2304/3375 (68.3%) of clinical versus 1554/3394 (45.8%) of preclinical students agreed with the statement that information from a pharmaceutical company brochure was useful (*P* < .001).

- Bias in information

A higher percentage of clinical than preclinical students had opinions about both the presence and absence of bias in pharmaceutical industry information from brochures or sales representatives. For example, more clinical than preclinical students believed that information in brochures is biased (979/3373, 29.0% versus 799/3380, 23.6%, *P* < .001), and more clinical students also thought that such information is not biased (363/3373, 10.8% versus 169/3380, 5.0%, respectively; *P* < .001). A similar proportion of preclinical and clinical students thought that information obtained at seminars was biased (764/3376, 22.6% versus 761/3354, 22.7%, respectively). We found that 7.2% (243/3395) of preclinical students and 18.0% (607/3376) of clinical students believed that one or more of the three forms of information was biased. In comparison, 3.5% (118/3395) of preclinical students and 8.1% (274/3376) of clinical students did not believe that there was any bias in any of the three forms of information from industry.

- Gift appropriateness

A substantial proportion of preclinical students thought it was appropriate to accept stationery (62.7%), a brochure (59.7%), or lunch (52.0%) or to attend a seminar (62.9%). A lower proportion of preclinical students thought it was appropriate to accept a textbook (42.8%). About 10% more clinical than preclinical students thought it was appropriate to accept each one of the five evaluated items. More than 20% of preclinical and clinical students thought it was inappropriate to accept a textbook. However, fewer students thought it was inappropriate to accept stationary (8.0% of preclinical and 6.9% of clinical students), a brochure (5.7% of preclinical and 3.6% of clinical students), lunch (12.3% of preclinical and 9.1% of clinical students), and a seminar (3.7% of preclinical and 2.6% of clinical students). Of students who perceived that they had received education on conflicts of interest with the pharmaceutical industry (*n* = 1675), there was no difference between the groups with and without exposure to a formal curriculum on drug promotion in the percentage of students who thought that every item from industry was appropriate.

- Influence on practice

More clinical students agreed that accepting stationery or lunch did not influence physicians’ practice; regarding lunch, 2391/3367 (71.0%) of clinical versus 2050/3385 (60.6%) of preclinical students held this view (*P* < .001). Only about 10% of students thought that accepting stationery or lunch influenced practice.

### Predictors of viewing gifts as appropriate

Table 4 shows the results of multivariate logistic regression analyses of factors associated with students’ attitudes toward the appropriateness of accepting gifts from the pharmaceutical industry.

**Table 4:** Multivariate predictors of students’ perception of gift appropriateness

Independent variables	Odds ratios (95% confidence intervals) for students’ perception of gift appropriateness				
	Type of gift or event				
	Stationery	Textbook	Brochure	Lunch	Seminar
Sex (male = 1)	0.92 (0.82–1.04)	1.06 (0.96–1.18)	0.95 (0.85–1.07)	1.00 (0.89–1.12)	0.91 (0.81–1.02)
Physician parent (yes = 1)	1.06 (0.93–1.20)	1.03 (0.92–1.14)	1.06 (0.95–1.20)	1.04 (0.92–1.17)	1.01 (0.89–1.13)
Received prior teaching on the physician-industry relationship (yes = 1)	1.08 (0.94–1.25)	1.09 (0.97–1.23)	1.27 (1.10–1.46)*	1.04 (0.91–1.20)	1.38 (1.20–1.59)*
Exposure to formal curriculum (yes = 1)	0.81 (0.69–0.95)*	1.06 (0.93–1.21)	0.95 (0.81–1.12)	0.93 (0.79–1.09)	1.04 (0.88–1.22)
Type of school (private = 1)	1.05 (0.93–1.20)	1.33 (1.19–1.48)*	0.91 (0.81–1.03)	1.17 (1.03–1.33)*	0.96 (0.85–1.08)
Interaction with pharmaceutical industry (ever interacted = 1)	1.67 (1.47–1.89)*	3.07 (2.66–3.54)*	1.90 (1.68–2.14)*	1.71 (1.52–1.94)*	1.31 (1.15–1.48)*
Informational value (useful = 1, not useful = 0)	–	–	2.56 (2.30–2.86)*	–	3.23 (2.89–3.62)*
Bias in information (not biased = 1, biased = 0)	–	–	3.50 (2.64–4.65)*	–	4.13 (3.13–5.46)*
Influence on practice (not influential = 1, influential = 0)	6.57 (5.86–7.38)*	–	–	7.61 (6.79–8.55)*	–

*P-value < .05

Multivariate logistic regression analysis (simultaneous method)

For all statistical analyses, *P*-values were 2-tailed and those less than .05 were considered statistically significant

Perception of the appropriateness of gifts was dichotomized into appropriate/somewhat appropriate and neutral/somewhat inappropriate/inappropriate, with the latter category as the reference

Independent variables were dichotomized as follows, with the second category in each case considered to be the reference: national/public and private for type of school; agree/somewhat agree and neutral/somewhat disagree/disagree for “informational value”; not biased/not very biased and neutral/somewhat biased/biased for “bias in information”; not influential/not very influential and neutral/somewhat influential/influential for “influence on practice”

Prior acceptance of any item, the influence on practice of accepting stationery or lunch, and the informational value and bias in information in a brochure or a seminar were positively associated with students’ perceptions of gift appropriateness. Exposure to a formal curriculum on drug promotion was negatively associated with students’ perceptions of appropriateness, but only for stationery (OR: 0.81, 95% CI: 0.69–0.95, *P* = .01). The perception of having received prior teaching about the physician–pharmaceutical industry relationship was associated with perceived appropriateness of accepting a brochure (OR: 1.27, 95% CI: 1.10–1.46, *P* < .001) and attending a seminar (OR: 1.38, 95% CI: 1.20–1.59, *P* < .001). Attending a private school was associated with perceived appropriateness of accepting a textbook (OR: 1.33, 95% CI: 1.19–1.48, *P* < .001) and lunch (OR: 1.17, 95% CI: 1.03–1.33, *P* = .01). Sex was not associated with perceived appropriateness of accepting any type of gift.

## Discussion

This study investigated Japanese medical students’ interactions with and attitudes toward the pharmaceutical industry. Interaction with the industry and a sense that the industry had minimal influence on practice were associated with students’ perceptions that gift acceptance was appropriate. Students with exposure to a formal curriculum on drug promotion were more likely to think that it was appropriate to accept stationery, but not a textbook, a brochure, lunch, or a seminar.

More than 90% of Japanese clinical students had accepted stationery or lunch and 80% had attended a sponsored seminar. Further, interactions with the pharmaceutical industry were reported more commonly by clinical students than by preclinical students; fewer than 30% of the latter had had such interactions. Our observations are consistent with recent reports from other countries on the overall frequency of interactions [13, 26] and the trend toward higher frequencies in clinical students [20, 23, 26, 27]. The higher rate at which clinical students in this study interacted with the industry was similar to the pattern seen in Japanese physicians in a previous survey, in which 96% of respondents received stationery, and 80 and 93% attended industry-sponsored educational events inside and outside the workplace, respectively [19].

A survey at Washington University in the United States demonstrated that by the end of their first year, one-third of students had accepted a meal offered by the pharmaceutical industry [28]. The present study showed that one in three preclinical students had received stationery and one in eight had accepted lunch. In Japan, two-thirds of schools include early clinical immersion at community clinics, community hospitals, or nursing homes in their first-year curriculum [29]. Our findings suggest that education about the relationship with industry is required before students begin clinical immersion programs.

In this study, 45.8 and 68.3% of preclinical and clinical students, respectively, thought that information in pharmaceutical company brochures was useful, which is within the range found in recent studies (30–70%) [13, 15, 17, 24]. We found that 20–30% of students perceived bias in information received from the industry, a rate much lower than the reported 70–90% of students in the United States who considered that sponsored grand rounds were biased [13, 17, 22, 24]. One possible reason for this difference is the low level of education on critical appraisal of drug promotion in Japan. Our survey of formal curricula on drug promotion (survey II) showed that only 6.8% (3/44) of schools in Japan had a formal curriculum teaching critical appraisal of drug promotion, as reported in our paper on formal curricula in Japan [30]. This contrasts with North American countries where 79% of schools had such curricula [31]. In the present study, 62.7% of preclinical and 71.8% of clinical students thought that accepting stationery was appropriate. These percentages are higher than those in recent studies in which approximately half of respondents considered it appropriate to accept a meal, book, or small gift [13, 15, 17, 22], and they are compatible with the findings of other studies showing that clinical students are more likely than preclinical students to consider it appropriate to accept industry gifts [32, 33]. Regarding the perception of the influence of gifts, fewer than 10% of Japanese students in this study believed that accepting stationery or lunch would influence their clinical practice. This proportion is much lower than reported in studies from other countries, which indicate that 25–70% of students thought that receiving gifts or food increased the chance that their fellow students would eventually prescribe the company’s drugs [13, 15, 17, 23–25]. In one previous survey, as few as 16% of Japanese physicians stated that gifts from sales representatives had an unfavorable impact on prescriptions [19], and students’ attitudes toward the influence of gifts may be interpreted as a reflection of the views of their instructors. The lack of education about drug promotion in Japan noted above may also have contributed to these lower percentages [30].

The multiple regression analysis showed that participation in physician–industry interactions contributed to students’ perceptions of the appropriateness of industry gifts. This is in accord with a previous study showing that exposure to physician–industry interactions led to positive attitudes to industry; for example, feeling grateful for having drug-related information provided by a sales representative (odds ratio = 3.0) [34]. Research has also shown that interaction with the industry reduces skepticism and hesitation and increases physicians’ confidence that they will not be influenced by these interactions [35, 36]. The association between physician–industry interaction and attitudes to the appropriateness of such interactions found here is consistent with associations reported in recent studies. Sierles et al. assessed the association between skepticism (measured by responses to items on informational value, bias in information, and influence on practice) and attitudes about the appropriateness of receiving gifts, and reported that more skeptical students were less likely to think that gifts were appropriate [13, 17]. The current study showed that attitudes to the influence of industry gifts on practice had higher odds ratios than did interactions, informational value, and bias in information. This indicates that the lack of awareness of influence on practice is a more powerful determinant of students’ perception that gift giving is appropriate than other variables. Educational programs that aim to increase awareness of these influences are likely to be more effective at changing students’ attitudes.

There was a difference in the percentage of students who answered that they were educated about physician–industry relationships and the percentage of students who had actually been exposed to a formal curriculum on this topic. This may be because medical students perceive informal discussions with their faculty instructors during clinical clerkships as education on this topic [26, 37, 38]. Students’ perception that they had received such teaching was positively associated with the view that it was appropriate to accept stationery, a brochure, or attend a seminar, indicating that the education that students believed they were exposed to generated favorable attitudes toward the pharmaceutical industry. This interpretation is supported by the lack of a difference in the perception of appropriateness between students with and without exposure to a formal curriculum among those who perceived that they had been educated about drug promotion. These results confirm that role modeling is an important component of the informal curriculum influencing students’ attitudes, as also indicated in recent studies [13, 14, 39].

This study has several limitations. Although the background characteristics of the respondents, such as sex, type of school, and age, did not differ from national statistics [29], only about half of all medical schools in Japan participated, raising concerns about sampling bias. Many schools declined to participate because of the survey contents; others may have accepted more financial support from the pharmaceutical industry or may have had negative views on educating students about the relationship with the industry. Second, the respondents may have expressed socially desirable responses despite anonymity and self-administration. Third, we did not confirm whether individual participants had actually participated in a formal curriculum. Instead, we asked school officers if the class students belonged to had participated in the program, and because the programs were all required courses, we considered the students to have been exposed. Fourth, the contents of the formal curricula varied; the effects of these programs could be assessed more accurately if their contents were standardized, e.g., based on specific guidelines [40]. Fifth, 188 of 7029 students (2.7%) returned partial responses and their data were therefore excluded from the analysis. However, an analysis of the whole data that included the partial responses produced results that were essentially unchanged. Sixth, this was a cross-sectional survey and we cannot infer causal relationships between interactions and attitudes. Finally, the results may not be generalizable to medical education in other settings. Some research suggests that cultural differences need to be taken into account when considering education on medical professionalism [41], as relationships with the pharmaceutical industry are likely to be influenced by social and cultural background. Drawing on experiences from Western countries is helpful when considering educational interventions and implementation of regulations, but such experiences may not always apply to East Asian countries because of different cultural backgrounds. The current study is especially helpful when considering educational interventions and implementation of regulations in the East Asian countries. Our findings may also be useful to inform research on educational interventions in other countries in which pharmaceutical sales are expanding.

Future curriculum reforms would benefit from additional studies that investigate when, by whom, and how students are informally educated about the relationship with the pharmaceutical industry and that examine the contents of such informal teaching and its influence on students’ attitudes and behaviors.

## Conclusions

We report here Japanese medical students’ interactions with and attitudes toward the pharmaceutical industry. We demonstrated an association between students’ interactions, skeptical attitudes, and perceptions of education on the physician–industry relationship, and students’ opinions about gift appropriateness. These results show the association between students’ lack of awareness of the potential influence of gifts and their perception of the appropriateness of gifts. The association between exposure to a formal curriculum on drug promotion and students’ perceptions of gift appropriateness was limited. There have been no efforts in Japan to establish professional guidelines on the physician–industry relationship. We hope that this study promotes discussion of the development of appropriate regulations.

## Abbreviations

CI: Confidence interval
OR: Odds ratio
PR: Pharmaceutical representative
SD: Standard deviation.
